# A Practical Treatise on Inflammation, Ulceration and Induration of the Neck of the Uterus; with Remarks on the Value of Leucorrhœa and Prolapsus Uteri as Symptoms of Uterine Disease

**Published:** 1847-04

**Authors:** 


ART. VI.—*A Practical Treatise on Inflammation, Ulceration and Induration of the Neck of the Uterus: with Remarks on the value of Leucorrhœa and Prolapsus Uteri as Symptoms of Uterine Disease.* By JAMES HENRY BENNET, M. D., Licentiate of the Royal College of Physicians; formerly House Physician (by concours) to the Hospitals St. Louis, Notre Dame de la Pitie, and la Salpetriere, Paris; formerly Vicepresident of the Parisian Medical Society, etc.; Graduate in the Faculty of Arts and in the Faculty of Sciences of the University of Paris. Philadelphia: Lea and Blanchard: 1847. 146 pages.

The author of this little volume has enjoyed peculiar advantages for making himself familiar with the interesting affections of which it treats. Seven years he was connected with the Paris Hospitals, three as a pupil and four as a medical functionary, during which time his attention was directed especially to uterine pathology. In the course of his investigations the truth was forced upon him that much as the diseases of the uterus had been studied, very much still remained to be elucidated. One point in particular, he says, attracted his attention, namely, "The nature, causes, and therapeutics of ulceration and induration of the neck of the uterus, the commonest of all uterine lesions." And in the work before us we have the fruits of his reflection and experience concerning these maladies.

It is in France that diseases of the female organs of generation are likely to be investigated with the greatest success. There, examinations by the *toucher* and by the speculum are submitted to continually, when the uterine organs are the seat of any disorder however slight. In our country and in England such examinations are not made, unless there be serious occasion for the step. What the result must be it is not difficult to divine. Our knowledge of uterine diseases must necessarily advance slowly, if we are left to the few opportunities afforded us of inspecting their seat and character. No doubt the modesty of our females is excessive, but, great as might be the utility to obstetric medicine, we could not desire to see it so far relaxed as to permit the use of the speculum in every case where disease of the uterus was felt or imagined. Dr. Bennet is naturally a strong advocate for such examinations, and he cites cases to show that women sometimes suffer long because their medical attendants, from excessive delicacy, neglect the only means by which they can become informed as to the true nature of the affection.

Chapter I treats of inflammation, ulceration, and induration of the cervix uteri in women who have not borne children. Having adverted to the fact that the cervix uteri in the healthy condition is perfectly soft, smooth and unctuous to the touch, and entirely free from pain on pressure, he remarks that when inflamed it will be found hotter than the lower part of the vagina, and destitute of its unctuous, greasy feel. When ulceration is set up, it may be recognised, according to most pathologists, "by its producing the sensation that a velvety surface would offer when the finger is passed lightly over it." But this peculiar sensation, Dr. Bennet has found, is not easily appreciated, and he is more disposed to rely upon the induration of the tissue underneath the mucous surface, which, according to his observation, will always be felt, and is very perceptible to the touch. He remarks—

"In the form of ulceration that we are now examining, the induration to which I allude is quite superficial, not extending to the central tissue of the uterine neck. It is merely a thickening of the ulcerated mucous membrane, and of the subcellular tissue, most perceptible at the circumference of the ulceration; yet it is easily appreciated by the finger of one who is accustomed to look for it, and to him it is a valuable symptom. This superficial induration is generally felt most distinctly at the edge of the uterine lips, where the mucous membrane passes into the cavity of the neck, and where, consequently, two mucous thicknesses are approximated by the folding of the membrane. Although I have found this symptom of great assistance in the diagnosis of ulcerations, I must confess, nevertheless, that it is not infallible. In the very first stage of ulceration, induration may not yet exist, while, on the other hand, the ulceration may heal and the superficial induration remain for a few days. When the inflammatory induration extends to the entire substance of the cervix, as it generally does if the ulceration exists in women who have had children, the superficial induration is necessarily lost in the general hardness. Pressure on the inflamed and ulcerated cervix will often, not always, occasion slight pain, which is never the case in the healthy state."

Such being the difficulty in arriving at a satisfactory diagnosis by means of the touch alone, Dr. Bennet considers it necessary to resort to the speculum in order to ascertain the exact state of things. The appearances presented under such circumstances are thus described:

"A certain quantity of mucoso-purulent matter is always found at the superior region of the vagina, even when the lining membrane of that organ is not inflamed; the cervix uteri is generally increased in size; but seldom so much so as not to be admitted into the cavity of an ordinary sized conical speculum, the one I generally use, and by far the most convenient and the least painful to the patient. The tumefaction is mostly greatest on the upper lip, which is the larger one of the two in the healthy condition; it is therefore often neecessary, in order to expose the orifice of the os, to raise the speculum towards the pubis, and by thus slightly pressing with the superior edge of the instrument on the anterior lip, to push it back and allow the inferior one to enter its cavity. Even if the cervix uteri is too large to be admitted at once into the speculum, by thus alternately depressing its different parts the entire organ may successively be brought fairly into view. When inflamed, the tumefied cervix presents a more or less intense red, glistening hue, instead of the pale, dull, whitish color which is natural to it. On its surface may frequently be seen small white or red vesicular or papular elevations, the result of distention of the mucous follicles or of their hypertrophy. Different forms of inflammation have been admitted by some writers, founded on this appearance, but without any practical utility whatever. When the mucous membrane is ulcerated, the glossy appearance of the membranous surface is lost, and a number of vascular granulations of a vivid red hue are seen covering the ulcerated region after the mucus has been wiped away with a pledget of lint—a necessary precaution. Sometimes the ulcerated surface appears raised above the adjacent level, while occasionally, on the contrary, it appears depressed. When the ulceration is at the entrance of the os uteri, it is often difficult to discover unless the uterine lips be slightly separated. There is generally a mass of semi-transparent mucus occupying the cavity of the os uteri. The ulceration may be so superficial and slight as to be scarcely perceptible, or extend over a considerable portion of the cervix. In many cases, the pressure of the edge of the speculum, or even of the pledget with which the mucus is wiped off, occasions a slight oozing of blood from the abraded or ulcerated surface. This also frequently occurs when patients thus affected expose themselves to intercourse—a fact of which they themselves are often cognisant. Menstruation is generally more painful than in the healthy state, owing to the temporary congestion of the uterus increasing the inflammatory irritation of the cervix. Indeed, the occurrence of the various symptoms of painful and difficult menstruation, when coupled with a leucorrhœal discharge, may be considered in most cases as pathognomonic of inflammation and ulceration of the cervix. Occasionally slight irritation of the urinary organs is present, giving rise to frequent desire to urinate. The annoyance and distress of mind which the local symptoms sometimes produce, coupled with the leucorrhœal discharge when it is abundant, may react more or less on the general health, and give rise to dyspepsia, palpitation, general weakness, etc."

To this description of inflammation and ulceration of the cervix are appended cases illustrative of the disorder. We copy one of these, as having interest in many respects:

"In Paris, as all who are acquainted with Parisian matters well know, the police is very severe and exercises great scrutiny and control over all persons who are not regularly domiciliated householders. In pursuance of this line of conduct, domiciliary visits are made at irregular periods, in the middle of the night, in the lower order of hotels or lodging houses, and also in those inhabited by students. This is a precaution rendered absolutely necessary by the irregularity of the lives of some of them, and by the circumstance of their congregating, to the number of fifty, a hundred, or more, in the favorite hotels of the 'Pays Latin.' When these '*descentes*,' as they are called, take place, every room is visited, and all persons whose passports are not found in order, as also all females, are forwarded to the prefecture of police. The following morning, the latter are generally sent to St. Lazare, (the hospital and penitentiary for unfortunates,) unless claimed by two respectable householders. These severe means are adopted partly with a view to the discovery and arrest of suspicious characters, and partly as a moral check. On one of these descentes, a young person named Jourg, eighteen years of age, was taken, and not having any friends, was detained by the police. In the course of a few days she was examined by the police medical authorities—a precaution usually adopted in these cases previous to being discharged—and was found by them to be laboring under slight ulceration of the os uteri.

"It was thought that the affection might be syphilitic, and as she was not an enrolled woman of the town, she was sent to a general hospital (l'Ourcine) and not to the infirmary of St. Lazare. The hospital physician kept her for a few days, and then, not considering her affection sufficiently severe to require further treatment, sent her back to the police. Here she was again examined by the police physician, who, finding that the ulceration had not been cured, sent her into M. Emery's wards at St. Louis, where she consequently came under my notice.

"On examination by the toucher, 4th July, 1843, the cervix uteri appeared small and soft, and there was a scarcely perceptible, very superficial and very circumscribed induration; no pain on pressure. The speculum showed the vagina to be narrow and of the natural hue unto very nearly its superior extremity, where it became rather red and injected. The cervix was small, about the size of the ungual portion of the medius finger; it was evidently congested, but soft, offering little or no resistance to pressure. On its anterior aspect there was a small abrasion about the size of a sixpence, covered with minute red granulations and a little semi-purulent mucus. There was no other mucoso-purulent discharge in the vicinity. The mucus issuing from the uterine orifice was perfectly transparent; no pain whatever in the loins or hypogastric region; no heat or burning sensations; no leucorrhœal discharge; health perfectly good. The patient said that had she not been told she was ill, she should not have thought that there was anything at all the matter with her. She stated that she had been brought up in the country; that eight months previously she had come to Paris, and had lived since her arrival by working as a semstress; that she had made acquaintance with a student, who had persuaded her to accompany him home to his lodgings a few days before she was seized by the police, and that it was the first and only time she had known any one—an assertion which the state of the organs tended to corroborate. She had menstruated for three years, had never experienced any leucorrhœal discharge whatever, and had always been in excellent health. M. Emery, the physician who at first examined her, told me that the lesion, which was very slight indeed, had increased since then; while she was at the Ourcine, she had been treated by emollient injections. It was therefore considered that these means were not sufficiently energetic, and the ulcerated surface was cauterized by the acid nitrate of mercury. Emollient injections and general baths were also resorted to.

"The tumefaction of the cervix and the ulceration increased under the influence of the first cauterization, (it was evidently rather too energetic,) but decreased under that of the second, third, and fourth, which were performed at intervals of six days.

"On the 5th of August the tumefaction and redness of the cervix had disappeared, and the ulceration was almost healed. Astringent injections were then alone used, and on the 15th of August she left, perfectly cured."

This disease is not unfrequently the cause of sterility, according to Dr. Bennet. He was assured by Emery, Gendrin, and Jobert, in Paris, that they had known many young married women who had remained barren from this cause, and had become pregnant as soon as cured.

There can be but one opinion, we are sure, respecting this work, and that is, that it is a most important monograph. We fear that success like that which seems to have attended the practice of the author will not always crown the efforts of other practitioners, but there is no reason to doubt that, guided by his treatise, they will oftener be correct in their diagnosis. It may be that one reason why his remedies have not availed as much in other hands as his own is, that they have not been applied with as much perseverance. Cases of this description demand of the practitioner great patience. Many, no doubt, fail of recovery because the patient or her medical attendant grows weary in the use of the appropriate means.

